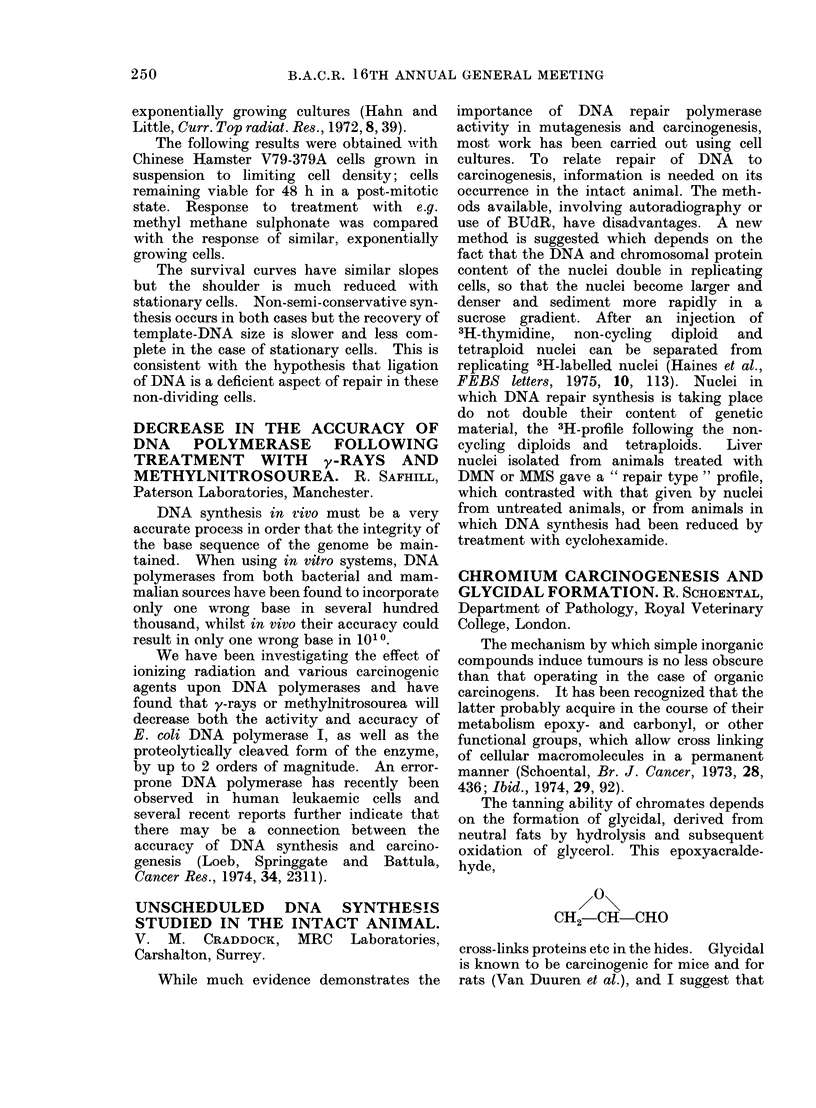# Proceedings: Decrease in the accuracy of DNA polymerase following treatment with gamma-rays and methylnitrosourea.

**DOI:** 10.1038/bjc.1975.188

**Published:** 1975-08

**Authors:** R. Safhill


**DECREASE IN THE ACCURACY OF DNA POLYMERASE FOLLOWING TREATMENT WITH γ-RAYS AND METHYLNITROSOUREA.** R. SAFHILL, Paterson Laboratories, Manchester.

DNA synthesis *in vivo* must be a very accurate process in order that the integrity of the base sequence of the genome be maintained. When using *in vitro* systems, DNA polymerases from both bacterial and mammalian sources have been found to incorporate only one wrong base in several hundred thousand, whilst *in vivo* their accuracy could result in only one wrong base in $10^{10}$.

We have been investigating the effect of ionizing radiation and various carcinogenic agents upon DNA polymerases and have found that γ-rays or methylnitrosourea will decrease both the activity and accuracy of *E. coli* DNA polymerase I, as well as the proteolytically cleaved form of the enzyme, by up to 2 orders of magnitude. An error-prone DNA polymerase has recently been observed in human leukaemic cells and several recent reports further indicate that there may be a connection between the accuracy of DNA synthesis and carcinogenesis (Loeb, Springgate and Battula, *Cancer Res.*, 1974, **34**, 2311).